# Sequences of Reverse Transcribed Brain DNA Are Modified by Learning

**DOI:** 10.3389/fnmol.2020.00057

**Published:** 2020-04-28

**Authors:** Antonio Giuditta, Joyce Casalino

**Affiliations:** ^1^Accademia di Scienze Fisiche e Matematiche, Naples, Italy; ^2^Biology Department, Federico II University, Naples, Italy

**Keywords:** brain metabolic DNA (BMD), DNA sequences, learning, synaptosomes, reverse transcription

## Abstract

Brain metabolic DNA (BMD) is continuously synthesized by reverse transcription in presynaptic synaptosomes and astroglia, and is partly transferred to nuclei after acquiring the double stranded configuration. Synthesis and turnover of BMD are markedly dependent on brain activity, as shown by circadian oscillations, environmental enrichment and impoverishment, and a variety of learning protocols. In rodents learning a two-way active avoidance task, BMD synthesis doubles, thus raising the possibility that sequences of learning BMD may differ from control BMD. The hypothesis has now been examined by sequencing cytoplasmic BMD. The present data indicate that most high-quality mapped BMD fragments hosting more than seven sequences are present in all mice. Three of them are exclusively present in learning BMD and four in control BMD. In addition, the annotated genes closest to them are mostly involved in modulating synaptic activity. The data support the conclusion that learning BMD sequences encode brain responses to the modified environment.

## Introduction

Brain metabolic DNA (BMD) is continuously synthesized in rodents and undergoes a markedly modulated turnover dependent on brain activity. Indeed, its synthesis and degradation (Perrone et al., 1982) is modified by stress (Giuditta et al., 1978), strain (Papa et al., 1995), circadian oscillations (Grassi Zucconi et al., 1988b), enriched and impoverished environment (Grassi Zucconi et al., 1988a, 1990), a variety of learning protocols (Reinis, 1972; Ashapkin et al., 1983; Scaroni et al., 1983; Giuditta et al., 1986; Ivashkina et al., 2012) and post-trial sleep (Giuditta et al., 1985; Ambrosini et al., 1988; Langella et al., 1992). BMD synthesis is also modulated by long-term habituation and potentiation (Sadile et al., 1991, 1995c) and controlled by the dorsal noradrenergic bundle (Sadile et al., 1995a, b). BMD largely localizes in glial cells but is also present in neurons, often in peri-nucleolar regions (Sjöstrand, 1965; Watson, 1965; Reinis, 1972).

The intriguing properties of brain DNA previously outlined in a book chapter (Giuditta, 1983) have been recently updated in a recent review devoted to BMD (Giuditta et al., 2017). In addition, BMD behavior in cesium gradients have demonstrated the *in vivo* BMD origin by cytoplasmic reverse transcription (Giuditta and Rutigliano, 2018), thus confirming *in vitro* data supporting BMD synthesis by RNA dependent DNA polymerase (Salganik et al., 1983). The former data concerned [^3^H]thymidine-labeled BMD from brain subcellular fractions and purified nuclei after incorporation times ranging from 30 min to several weeks. These results have been fully confirmed by immunofluorescent analyses, that have also showed BMD reverse transcription in presynaptic synaptosomes and astroglial processes (Cefaliello et al., 2019; Prisco et al., 2019).

The twofold increase in BMD synthesis in rodents learning avoidance tasks (Reinis, 1972; Ashapkin et al., 1983; Scaroni et al., 1983; Ivashkina et al., 2012) has suggested the hypothesis that learning BMD might differ from control BMD not only in content, but also in sequences. This possibility was in agreement with the BMD distribution in repetitive and non-repetitive DNA fractions determined in rats learning an appetitive task (Giuditta et al., 1986). Indeed, while the double stranded BMD of highly repetitive DNA fractions was markedly lower in learning rats than in control rats, the single stranded BMD of non-repetitive DNA fractions was markedly higher in learning rats than in control rats.

To compare BMD sequences of mice learning a two-way active avoidance task with those of control mice, cytoplasmic BMD was purified and analyzed by Illumina MiSeq program. Of the high-quality consensus regions displaying more than seven sequences (SCR) that were present in all mice (*n* = 1,005) three were exclusively present in learning mice, and four were exclusively present in control mice. In addition, annotated genes closest to these SCR are prevalently involved in modulating synaptic activity. The data support the hypothesis and suggest that BMD role concerns the encoding of adaptive, experience-dependent brain responses.

## Methods

### Preparation of Cytoplasmic Fractions

Soon after the training session, each learning mouse and the corresponding control mouse were killed by dislocation; brains were dissected and freed of extraneous material at ice temperature; and cerebral hemispheres were homogenized with a Dounce homogenizer in 9 ml of ice-cold isotonic medium (0.32 M sucrose, 10 mM Tris-Cl pH 7.4). Homogenates were centrifuged in an Eppendorf table centrifuge (4°, 1,000 g, 4 min) to sediment the nuclear fraction that was discarded, while the supernatant fraction was centrifuged at higher speed for a longer time (4°, 20,000 g, 30 min) to sediment the cytoplasmic fraction that was stored at −80°. This procedure was adopted since previous experiments showed that post-nuclear supernatant was completely free of nuclei easily identified by Hoechst 33228 staining.

### Purification and Sequencing of Cytoplasmic BMD by BMR Genomics, Padua, Italy

BMD was purified from the frozen cytoplasmic sediment of each mouse with a Qiagen kit, and 2 × 300 bp sequences obtained by Illumina MiSeq procedure. Fragments were also assembled with SPAdes 3.7 procedure for Illumina paired-end reads to yield contig sequences whose maximum size attained 16,426 bp in C1, C2 and L2, and 17,066 in L1. Using BWA 0.7.13-r1126 program, all contig sequences were unambiguously, correctly mapped in each autosomic chromosome (mm10), X and Y chromosomes, and mitochondrial chromosome. The longest sequences mapped on mitochondrial chromosomes, sexual chromosomes, and several autosomic chromosomes.

As shown in Figure 2, the electrophoretic migration of BMD confirmed the cytoplasmic prevalence of learning BMD, in full agreement with the prevailing [^3^H]thymidine-labeled BMD or bromodeoxyuridine-labeled BMD in rodents learning the avoidance task (Reinis, 1972; Ashapkin et al., 1983; Scaroni et al., 1983; Ivashkina et al., 2012). Figure 2 also shows BMD size of 16–18 kbp, in agreement with the maximum size attained by contig sequences.

### Data Reported in All Tables and Figures

They were obtained by Dr. Claudia Angelini (Istituto per le Applicazioni del Calcolo “M. Picone,” Napoli, Italy) by using the following procedures. Aligned sequences were first converted from the original BAM files to BED files using bedtools^1^, and then analyzed using a customized R script^2^. Sequences mapping to non-canonical and mitochondrial chromosomes were filtered out, those with multiple mapping positions removed, and sequences with a mapping quality of at least 10 retained. Sequences with inconsistent alignment of the two pairs in terms of chromosome, orientation, and distance were filtered out, allowing a maximum distance of 2,000 bp between pairs. Finally, the two mates were joined into a single fragment and single fragments were added. The analysis was independently performed on each mouse sample.

In addition, sequences of all samples were combined into a single list of raw sequences and Consensus Regions were obtained by superimposing more sequences using the Genomic Ranges package^3^ (Lawrence et al., 2013), and allowing a maximum gap of 2,000 bp to combine sequences into larger regions. To easily identify potential artifacts, consensus regions were markedly depending on their overlap with blacklist regions available for mm10^4^. To quantify the number of sequences of each sample mapping within each consensus regions, featureCounts from Rsubread package^5^ (Liao et al., 2014) was used. Consensus regions that exhibited more than seven mapped sequences after summing up all samples were denoted significant consensus regions.

Gene Annotation was performed by the annotatePeakInBatch function from ChIPpeakAnno package^6^ (Zhu et al., 2010) using the TxDb.Mmusculus.UCSC.mm10.knownGene^7^ as gene annotation database and ignore.strand = TRUE. The org.Mm.eg.db database was used to convert Entrez Gene identifiers and Gene symbol. Fasta sequences of significant consensus regions were retrieved using the BSgenome package^8^ and the BSgenome.Mmusculus.UCSC.mm10 database^9^. Gene Ontology was performed using the getEnrichedGO function of the ChIPpeakAnno package on the genes annotated with respect to the significant consensus regions, using as parameters maxP = 0.01, minGOterm = 10, multiAdjMethod = “BH,” condense = TRUE. Gene Ontology analysis was also performed using the gProfileR package^10^.

## Results

### Training Mice for a Two-Way Active Avoidance Task

The experiment concerned two male Bl63/c57 mice aged 2 months that were identified as L1 and L2, while their control mice were C1 and C2. At about 9.15, one mouse was transferred from its home cage to the shuttle-box in which it could move from one side to the other through a small opening in the separating division. The shuttle-box was placed in a darkened room After 15 min familiarization with the new environment, the mouse was exposed to a 30 min training period that included 60 training cycles lasting 30 s. Each cycle started with the turning on of a white light lasting 6 s. After the first 3 s, a foot-shock of mild intensity lasting 3 s was administered through the metal rod floor; in the following 24 s the shuttle-box remained dark. Foot-shocks were not delivered or were stopped whenever the mouse run to the other side. If the response occurred during the foot-shock, it scored an escape elicited by the foot-shock; if it occurred before the foot-shock, it scored an avoidance. The mouse was exposed to three training periods separated by rest periods of 30 min during which the mouse remained in the darkened shuttle-box. Mice were considered to have learned the task when most responses were avoidances. Since training cycles were contiguous, the mouse was compelled to learn that to avoid the foot-shock it had to move to the other side of the shuttle-box from which it had just run away. This additional difficulty contributed to extending the number of training periods to three.

**FIGURE 1:**
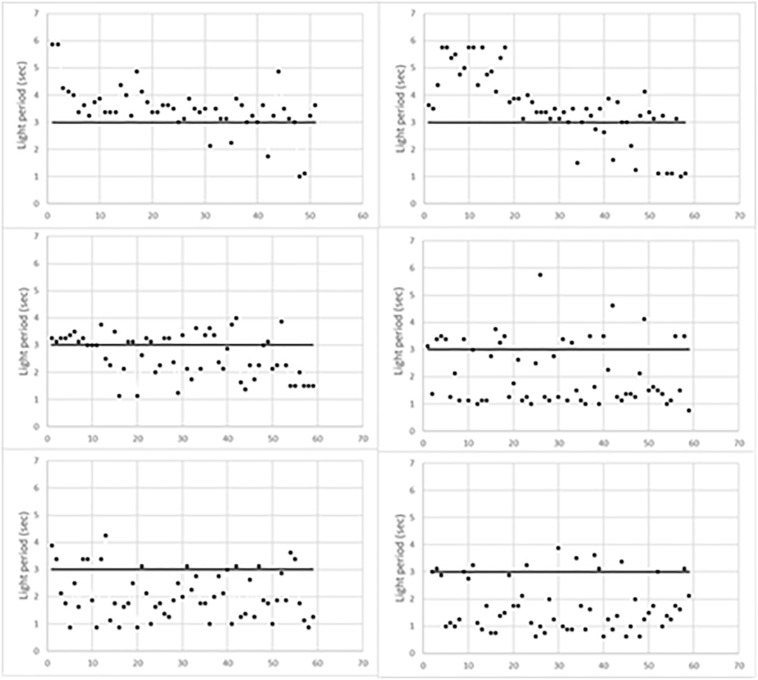
Behavioral responses of learning mice during the three training periods. L1, **left** column; L2, **right** column. The first training period is the top one.

Figure 1 indicates the time of occurrence of mouse behavioral responses with respect to the initial 3 s of light (horizontal line). Responses above the horizontal line occurred during the foot-shock period, thus scoring escapes; conversely, those below the horizontal line occurred before the foot-shock, thus scoring avoidances. As expected, escapes prevailed during the first training period while avoidances progressively prevailed in the last two periods. Figure 1 also shows that the timing of behavioral responses differed in each trained mouse. Indeed, in the first training period, most escapes of L1 occurred soon after the start of the foot-shock. Conversely, in L2, they occurred during the last 2 s of the foot-shock. In addition, in the second training period, most L1 avoidances occurred during the third second of light, that is just before the delivery of the foot-shock, while L2 avoidances took place earlier, during the first 2 s of light. Differences were less evident in the third training period but L1 avoidances were widely scattered in time while most L2 avoidances occurred in the first 2 s of light.

**FIGURE 2:**
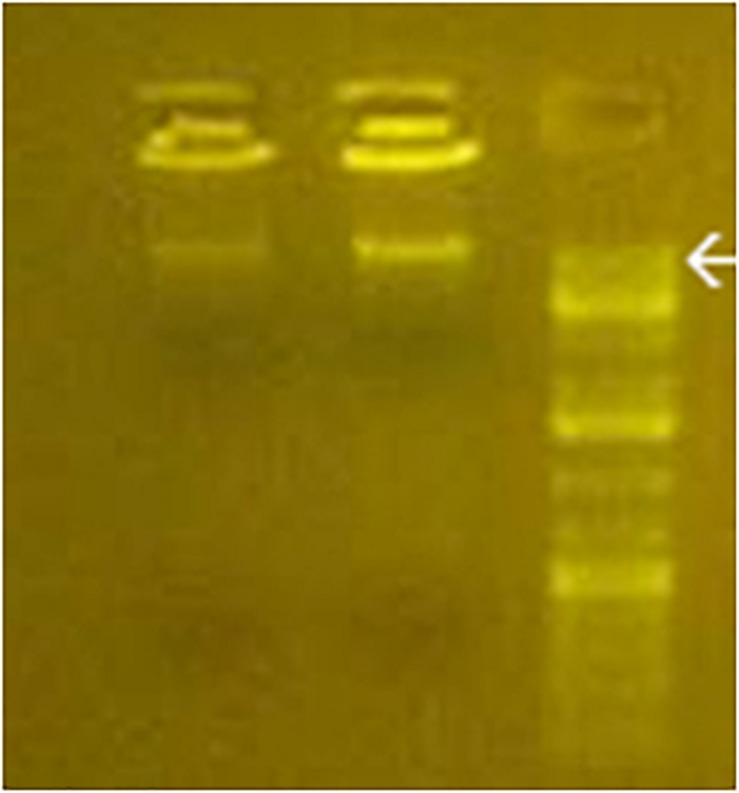
Gel electrophoresis of purified cytoplasmic BMD from C1 and L1 (left and middle line). DNA standards are shown in the right line. Arrow, 20 kbp.

### Preparation and Chromosome Alignment of High-Quality Sequences

Sequences mapped in multiple chromosomal positions, low-quality sequences, and sequences aligned to mitochondria and non-canonical chromosomes were discarded. After additional filtering out of chromosomal inconsistencies and paired mates resting at more than 2 kbp, high-quality (HQ) sequences were retrieved and separated in fragments containing both mapped mates (R1 + R2) or either mate. The former fragments numbered 157,965 and 135,974 in L1 and C1, and 197,159 and 178,994 in L2 and C2. On the other hand, R1 and R2 fragments exhibited markedly lower numbers, respectively, 4,422 (R1) and 1,620 (R2). When compared to raw sequences mapping to chromosomes that, respectively, numbered 186,280 and 167,733 in L1 and C1, and 226,818 and 205,733 in L2 and C2, all HQ mapped fragments displayed percent values higher than 80%. Specifically, they attained 84.7 and 81.1% in L1 and C1, and 86.9 and 87.0% in L2 and C2.

**FIGURE 3:**
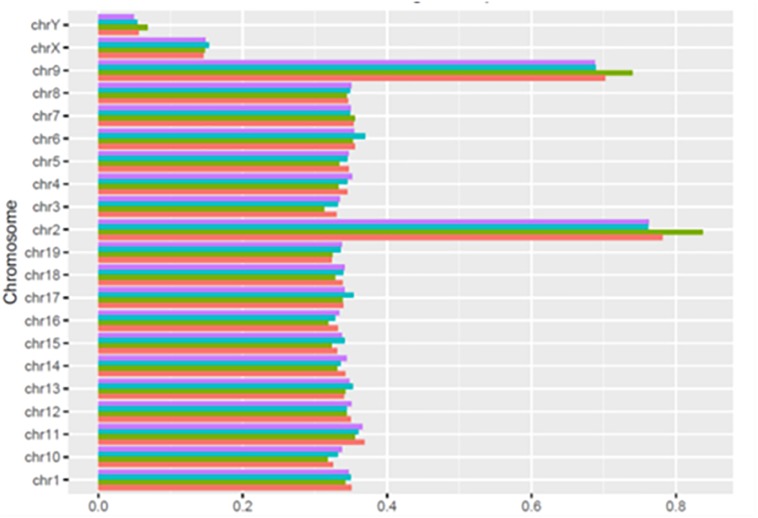
Normalized distribution of HQ mapped fragments in chromosomes of learning and control mice. C1, red; L1, green; C2, light blue; L2 purple.

As shown in Figure 3, the number of HQ mapped fragments normalized for chromosome size and sequencing depth, was close to 0.35 kilobase per million mapped sequences (FPKM) in most chromosomes, but the number doubled in chromosomes 2 and 9 (respectively, 0.68 and 0.83), and became conspicuously lower in chromosomes X and Y (respectively, 0.14 and 0.06). Notably, in all chromosomes, differences between learning and control mice were minimal. The higher FPKM values of chromosomes 2 and 9 were mostly due to sequences intersecting black regions unlikely to be trusted (Table 4)^11^.

### Preparation and Properties of Significant Consensus Reasons

**FIGURE 4:**
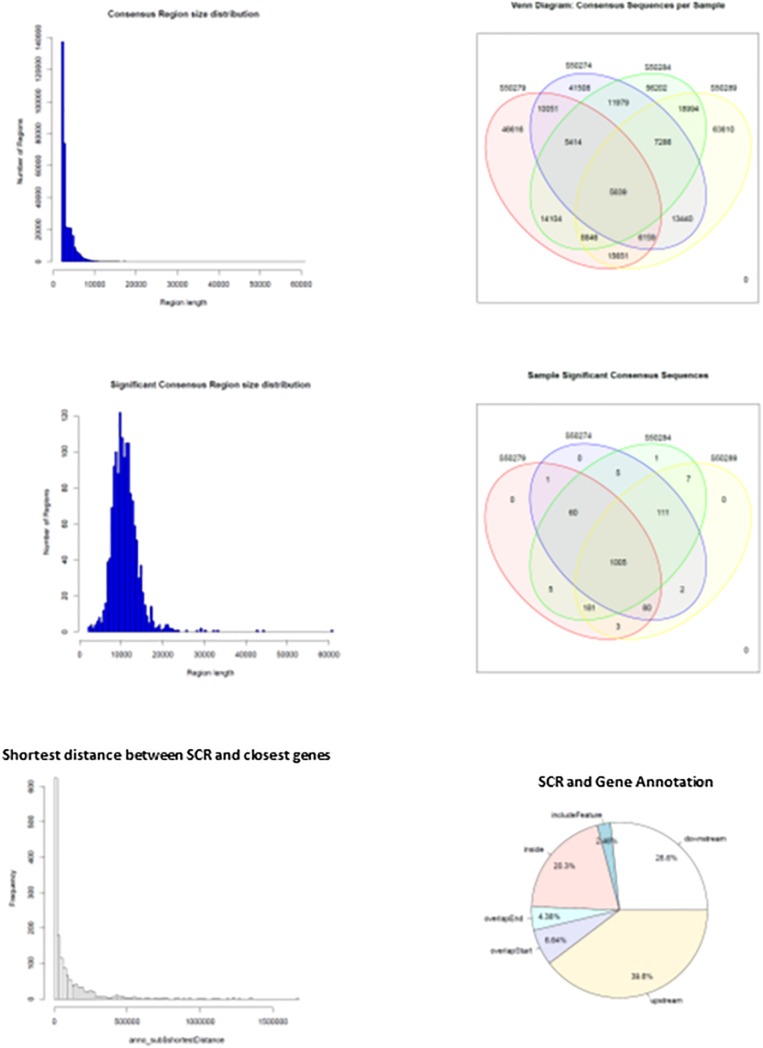
Main features of consensus regions (CR) and significant consensus regions (SCR). Top left panel, CR size distribution; top right panel, CR distribution in learning and control mice. Middle left panel, SCR size distribution; middle right panel, SCR distribution in learning and control mice. Bottom left panel, SCR distance from closest genes; bottom right panel, SCR position with respect to closest gene regions.

When HQ raw fragments present in all mice (*n* = 670,092) were superimposed to allow merging of overlapping regions, their number remained relatively high (*n* = 518,788), indicating that the number of merged regions was low and sample-specific raw fragments prevailed. Hence, merging of overlapping regions was extended by 2 kbp to allow overlapping of nearby sequences. This operation yielded a much higher number of merged regions that were identified as consensus regions (*n* = 325,899). They mostly exhibited sizes close to 2–3 kbp but also larger (Figure 4, upper left panel), and their distribution in learning and control mice (Figure 4, upper right panel) demonstrates that most were selectively present in each mouse: 46,616 and 41,508 in L1 and C1; 63,810 and 56,202 in L2 and C2, indicating a slight prevalence in learning mice (respectively, 12% in L1, and 13.5% in L2). On the other hand, consensus regions only present in both learning mice (15,651) and in both control mice (11,979) revealed a larger prevalence in learning mice (30.6%). Nonetheless, the number of these mapped sequences was limited to a few sequences and often to a single sequence, suggesting a possibly technical effect.

The results suggested that the number of mapped sequences could be used as a criterium apt to select more reliable consensus regions. Accordingly, using an empirical threshold, consensus regions exhibiting more than seven mapped sequences (high read coverage) were selected and identified as significant consensus reasons (SCR; *n* = 1,461) that were examined for properties and distribution in learning and control mice. SCR predominantly exhibited sizes close to 10 kbp but also larger (Figure 4, middle left panel), and were mostly present in all mice (*n* = 1,005, that is 69% of their total number; Figure 4, middle right panel). Notably, the genes closest to the latter SCR were identified and their role annotated (mm10), additional variables including SCR position with respect to closest genes (upstream or downstream), distances separating them, and overlapping SCR transcription (starting/ending site, inside, or including). The bottom panels of Figure 4 show the distribution of the distances between these SCR and their closest genes (left panel), and SCR positions with respect to gene regions (right panel). They highlight that the majority of SCR are relatively close to their genes, and that about one third is present within their closest gene.

### SCR Selectively Present in Learning and Control Mice

Three SCR were exclusively present in learning mice (Table 1, upper region), and four additional SCR were exclusively present in control mice (Table 1, lower region). Additional features include the chromosome number and position of SCR, its size and number of reads, and the identification of their closest gene (name, position and distance). Their largely prevalent lack of any intersection with the unreliable regions of the black list is also shown.

**TABLE 1:** SCR only present in learning or in control mice.

	**Position**		**Reads**		**Nearest gene**		
**chr**	**Start-end**	**Size**	**L1**	**L2**	**Name**	**Location**	**Distance**
**SCR only present in learning mice**							
5*	75345618–75356420	10803	5	4	Mir7025	Downstream	168787
7*	112685356–112697039	11684	4	4	Teadl	Inside	6038
8*	6034563–6034635	9073	4	4	Slcl0a2	Upstream	−929212
	**Position**		**Reads**		**Nearest gene**		
**chr**	**Start-end**	**Size**	**C1**	**C2**	**Name**	**Location**	**Distance**
**SCR only present in control mice**							
1**^∧^**	24610806–24614400	3595	20	10	Col19a1	Upstream	−23334
5*	117104229–117115931	11703	5	3	Suds3	Inside	11884
8*	32136497–32145198	8702	4	5	Nrgl	Inside	748300
16*	47038570–47046847	8278	3	5	Nectin3	Upstream	−540045

* Non-intersecting black list regions; ^∧^intersecting black list region.

The genes closest to the three SCR exclusively present in learning mice include Mir7025 which modulates post-transcriptional gene expression by acting on mRNA stability and translation; TEAD1 which regulates RNA Pol II and DNA binding; and SLC10A2 which is a sodium symporter involved in Alzheimer disease of US African people. The related SLC10A4 gene encodes a synaptic vesicle protein also involved in Alzheimer disease.

Conversely, the genes closest to the four SCR exclusively present in control mice include COL19A1 which is involved in developmental processes by encoding a collagen protein differently expressed in amyotrophic lateral sclerosis; NECTIN3 which is likewise involved in development and encodes an immunoglobulin-like cell adhesion molecule linking receptor 1 of corticotropin-releasing hormone to stress-induced memory deficits; SUDS3 which modulates cell processes and is member of chromatin remodeling complexes; and GM8179 which is a lncRNA gene.

### SCR Prevailing in Learning or in Control Mice

Since behavioral response times differed in learning mice (Figure 1) and were consequently likely to condition different BMD sequences, read number between learning and control mice might not be the same in both couples but might slightly differ. Indeed, on several occasions the lack of reads in the control mouse of one couple was associated with the presence of only a single read in the control mouse of the other couple. Furthermore, in most occasions learning mice exhibited a comparable number of reads. These conditions concerned 28 additional SCR which were identified as prevailing in learning mice (Table 2).

**TABLE 2:** SCR only prevailing in learning mice.

	**Position**			**Reads**				**Nearest gene**		
**chr**	**Start**	**End**	**Size**	**L1**	**C1**	**L2**	**C2**	**Name**	**Location**	**Distance**
**SCR prevailing in learning mice**										
1*	167644307	167661315	17009	4	1	3	0	Lmxla	Upstream	-44930
2*	13116052	13126537	10486	4	1	4	0	Clql3	Upstream	-104246
2*	106666249	106674772	8524	4	1	3	0	BB218582	Upstream	-15885
3*	128983523	128998428	14906	5	1	5	0	Pitx2	Upstream	-216355
4*	59379089	59390352	11264	3	1	4	0	Susdl	Inside	59544
4*	107476906	107490923	14018	4	0	4	1	Glisl	Inside	42315
4*	114836582	114848591	12010	4	1	4	0	Gm12830	Inside	14860
5*	75345618	75356420	10803	5	0	4	0	Mir7025	Downstream	168787
5*	106320627	106332164	11538	4	0	3	1	Gm32921	includeFeature	-713
5*	149577160	149593935	16776	4	0	6	1	Gml5997	Upstream	-39130
6*	31584756	31597231	12476	4	0	4	1	Gm6117	Downstream	9789
6*	57773994	57783599	9606	4	1	3	0	Voppl	Inside	51165
7*	47284318	47300122	15805	5	1	3	0	Mrgpra9	Upstream	-31470
7*	112685356	112697039	11684	4	0	4	0	Teadl	Inside	6038
7*	142789171	142798244	9074	4	0	3	1	Ins2	Upstream	-45790
8*	6034563	6043635	9073	4	0	4	0	Slcl0a2	Upstream	-929212
8*	87714627	87725567	10941	4	1	3	0	4933402J07Rik	Downstream	150774
9*	25048341	25056521	8181	4	0	3	1	E130101E03Rik	Downstream	74133
9*	30618769	30628064	9296	3	1	4	0	Snxl9	Downstream	191661
9*	30647312	30656964	9653	4	1	3	0	Snxl9	Downstream	220204
12*	61836093	61843609	7517	3	0	4	1	Lrfn5	Inside	312943
13*	104860143	104872455	12313	4	0	4	1	Shisal2b	OverlapStart	3750
14*	13597983	13606696	8714	4	0	3	1	Sntn	Upstream	-72893
14*	26737928	26744757	6830	3	0	4	1	Dnahl2	Inside	44654
14*	62928212	62939968	11757	4	1	4	0	Defb48	Downstream	56298
17*	26301197	26313647	12451	3	1	5	0	Luc7l	Downstream	48301
19*	28473658	28482623	8966	4	1	3	0	D930032P07Rik	Upstream	-204566
19*	45254631	45263023	8393	4	0	3	1	Lbxl	Upstream	-18819

* Non-intersecting black list regions; ^∧^, intersecting black list regions.

Likewise, the analogous identification of SCR prevailing in control mice (Table 3) showed that 13 SCR exhibited a comparable behavior, thus highlighting SCR prevailing in control mice.

**TABLE 3:** SCR only prevailing in control mice.

	**Position**			**Reads**				**Nearest gene**		
**chr**	**Start**	**End**	**Size**	**L1**	**C1**	**L2**	**C2**	**Name**	**Location**	**Distance**
**SCR prevailing in control mice**										
1^A^	24610806	24614400	3595	0	20	0	10	Coll9al	Upstream	-23334
1*	117953105	117961891	8787	1	3	0	5	Tsn	Downstream	358357
4*	156339582	156344510	4929	0	4	1	3	Vmn2rl23	Downstream	8447
5*	117104229	117115931	11703	0	5	0	3	Suds3	Inside	11884
8*	32136497	32145198	8702	0	4	0	5	Nrgl	Inside	748300
8*	90812733	90825659	12927	0	3	1	5	Gml9935	includeFeature	4660
9*	116267288	116276523	9236	1	4	0	3	Gm4668	Downstream	78108
11*	49211342	49221100	9759	1	5	0	3	Zfp62	overlapEnd	8050
11*	63140037	63146862	6826	0	4	1	3	Pmp22	Inside	11055
12*	69053179	69061389	8211	1	3	0	4	Rps29	Downstream	106007
14*	8332910	8344033	11124	0	3	1	4	Faml07a	Upstream	-14887
15*	85520397	85532653	12257	0	3	1	5	7530416GllRik	Upstream	-17170
16*	47038570	47046847	8278	0	3	0	5	Nectin3	Upstream	-540045

^∧^Intersecting black list regions; * non-intersecting black list regions.

### Number of SCR Reads in Chromosomes

As shown by Tables 1–3, SCR read number was mostly low, but considerably higher values were present in some chromosomes. As shown in Table 4, read numbers up to about 20 were present in several positions of chromosomes 4, 6, 7, 12, and 13, and values ranging between 20 and 60 reads occurred in chromosomes 5, 11, and 17. Moreover, still higher values ranging from hundreds to thousands reads were displayed by several positions of chromosomes 2, 6, 9, and 14, while the highest numbers were present in chromosomes 2 and 9, in agreement with the normalized chromosomal distribution of HQ fragments exhibiting twofold higher values only in these two chromosomes (Figure 3).

**TABLE 4:** Highly expressed SCR.

	**Position**	**Reads**			
**chr**	**Start-end**	**L1**	**C1**	**L2**	**C2**
**Highly expressed SCR**					
1^∧^	88211334–88254196	19	22	27	32
2^∧^	98661228–98669875	14360	10690	14881	13618
4^∧^	146474017–146503055	16	11	17	18
4^∧^	147411950–147435370	13	16	14	14
4^∧^	3049000–3072637	17	15	17	14
5^∧^	14986667–15047578	28	35	33	27
6^∧^	15457434–15490469	15	20	24	19
6^∧^	103648040–103650294	316	253	332	343
7^∧^	15668249–15698658	24	17	15	19
9^∧^	2998999–3043223	7032	5364	7274	6662
9^∧^	35302970–35307195	744	593	823	748
11^∧^	3180554–3201063	49	34	56	52
12^∧^	3108865–3112466	106	98	98	82
12^∧^	67056921–67063555	20	13	19	22
13^∧^	119594887–119606103	15	12	24	18
14^∧^	19412829–19420664	118	81	109	107
17^∧^	13539979–13554197	42	12	28	22
17^∧^	39841996–39849337	27	16	25	26
X^∧^	76595451–76600138	16	7	13	21

****^∧^****Intersecting black list regions.**

Read number was higher in L1 with respect to C1 in chromosome 2 (by 34%), in two positions of chromosome 9 (respectively, by 31 and 25%), and in chromosome 17 (by 350%). A markedly lower prevalence occurred in L2 with respect to C2 (respectively, by 9, 9, 10, and 27%). However, all these SCR intersected unreliable regions belonging to the black list.

### Nature of Genes Closest to SCR Present in All Mice

The annotation of genes closest to these SCR has been obtained using the getEnrichedGO function of ChIPpeakAnno package (Zhu et al., 2010). They indicate that a large majority modulates dendritic and synaptic activity involved in brain plastic processes. An example is provided by the 39 genes listed in Table 5. In addition, as shown in Table 1 and the related text, genes closest to the seven SCR exclusively present in learning or in control mice modulate protein or RNA synthesis. Their molecular role has been confirmed by gProfileR package analysis (see Methods) of the eight comparable genes listed in Table 6, most of which target RNA polymerase and DNA binding regions. Overall, the ontologies of genes closest to SCR present in all mice indicate that BMD encodes synaptic activity patterns adaptively modified by the subject’s experience and consequently worth saving as learned memory. The properties of the annotated genes closest to the 1,461 SCR present in all mice are shown in Supplementary Table S1.

**TABLE 5:** SCR cellular targets.

**GO ID**	**Go term**
**SCR cellular target**	
45202	Synapse
99055	Integral component of postsynaptic me.
98936	Intrinsic component of postsynaptic me.
99699	Integral component of synaptic me.
99240	Intrinsic component of synaptic me.
36477	Somatodendritic compartment
44456	Synapse part
98794	Postsynapse
120025	Plasma membrane bounded cell projection
97458	Neuron part
30425	Dendrite
45211	Postsynaptic me.
97447	Dendritic tree
42995	Cell projection
43005	Neuron projection
99061	Integral component of postsynaptic density me.
30424	Axon
98978	Glutamatergic synapse
98839	Postsynaptic density me.
99056	Integral component of presynaptic me.
98984	Neuron to neuron synapse
98889	Intrinsic component of presynaptic me.
99146	Intrinsic component of postsynaptic density me.
97060	Synaptic me.
44463	Cell projection part
120038	Plasma membrane bounded cell projection part
14069	Postsynaptic density
32279	Asymmetric synapse
42734	Presynaptic me.
99060	Integral component of postsynaptic specialization me.
99572	Postsynaptic specialization
98948	Intrinsic component of postsynaptic specialization me.
99634	Postsynaptic specialization me.
98590	Plasma membrane region
43235	Receptor complex
98793	Presynapse
34703	Cation channel complex
44304	Main axon
60076	Excitatory synapse

**GO ID, gene ontogeny identification; me, membrane.**

**TABLE 6:** SCR molecular targets.

**GO ID**	**Go term**
**SCR molecular target**	
1228	RNA polymerase II-specific DNA binding
977	RNA polymerase II sequence-specific DNA binding
43565	Sequence-specific DNA binding
1012	RNA polymerase II region DNA binding
5515	Protein binding
976	Sequence-specific DNA binding
3705	Transcription factor RNA polymerase II enhancer
30551	Cyclic nucleotide binding

**GO ID, gene ontogeny identification.**

## Discussion

The present experiments have examined the hypothesis that learning may modify BMD sequences. Since few mice could be sequenced by the financial support of AG pension, statistical significance cannot be provided, and results are to be regarded as belonging to a pilot experiment supporting the hypothesis and suggesting experimental conditions which could provide more incisive data.

To start with, data should be viewed in the light of BMD properties, chiefly those concerning its cytoplasmic origin by reverse transcription and the predominant origin in astroglial processes and presynaptic synaptosomes (Reinis, 1972; Cefaliello et al., 2019; Prisco et al., 2019). It is also of relevance that soon after the BMD synthesis as D/R hybrid, a significant fraction acquires the double stranded configuration and undergoes transfer to glial and neuronal nuclei. Furthermore, in control rats, [^3^H]thymidine-labeled BMD markedly increases in the first few hours but undergoes a marked loss (close to 50%) in the following few hours (Perrone et al., 1982). A comparable loss also occurs in the post-trial sleep of rats failing to learn a two-way active avoidance task (Giuditta et al., 1985; Ambrosini et al., 1988; Langella et al., 1992). Nuclear and cytoplasmic BMD are known to persist for weeks while undergoing a progressive decline (Giuditta and Rutigliano, 2018).

These features indicate that cytoplasmic BMD synthesized during the training session of learning mice and the comparable period of control mice also contains previously synthesized BMD. Conversely, cytoplasmic BMD lacks newly synthesized BMD that has been transferred to nuclei and to synaptosomes and other cell components that are known to sediment in the nuclear fraction. It follows that differences between learning and control mice based on the analysis of cytoplasmic BMD sequences only reflect a fraction of newly synthesized BMD and, in addition, are partly to be attributed to previously synthesized BMD. Additional interferences may be attributed to the different times behavioral responses have occurred in each learning mouse (Figure 1) since they clearly reflect the patterns of synaptic activity modulating BMD sequences. Clearly, more definite results are likely to be obtained by sequencing cytoplasmic and nuclear BMD exclusively synthesized during the training session, provided that it could be identified by the incorporation of an identifiable precursor. Unfortunately, available precursors ([^3^H]thymidine or bromodeoxyuridine) are known to interfere with sequencing procedures, thus suggesting the use of a different precursor.

An additional benefit of the latter suggestion might concern a more reliable identification of the control mate since previously synthesized BMD is not likely to be the same in learning and control mice differing in their previous experience despite their being exposed to the same environment. Hence, they are likely to interfere with the determination of sequence differences between learning and control BMD. The best theoretical solution would require comparing learning BMD with the control BMD of the same subject. Nonetheless, this apparently impossible solution could be attained by labeling learning BMD and comparing it with the unlabeled BMD of the same subject, thus comparing synaptic activities of the same subject exposed to a different experience.

An additional consideration regards the selective loss of learning BMD apparently elicited by the sequencing procedures. In fact, the marked prevalence of newly synthesized BMD in rodents learning an avoidance task has been repeatedly reported in the literature (Reinis, 1972; Ashapkin et al., 1983; Scaroni et al., 1983; Ivashkina et al., 2012), and also confirmed by the electrophoretic analysis of cytoplasmic BMD (Figure 2). Nonetheless, learning sequences aligned by BWA 0.7.13-r1126 program in BMR lab only exhibited a reduced prevalence with respect to control sequences (Giuditta and Casalino, 2018), and no prevalence occurred in HQ BMD fragments mapped to chromosomes (Figure 3). Since the loss selectively concerned learning BMD, it may not be excluded that learning BMD is partly endowed with features interfering with sequencing procedures.

Notwithstanding the above considerations, the present data demonstrate that sequences of learning BMD differ from those of control BMD. Indeed, three SCR are exclusively present in learning BMD, and four additional SCR are exclusively present in control BMD (Table 1). In learning SCR, the genes closest to two of them modulate transcription (Mir7025 and Tead1), and the gene closest to the third SCR (Slc10a2) is related to Alzheimer’s disease. On the other hand, in control SCR, the genes closest to two of them, respectively, encode the collagen of amyotrophic lateral sclerosis (Col19a1) and a cell adhesion protein (Nectin3), while the genes closest to the other SCR are, respectively, involved in chromatin remodeling (Suds3) and post-transcriptional regulation (lncRNA). Furthermore, 28 additional SCR prevail in learning mice, mostly by three reads, and more than half of them are positioned at a distance lower or close to 50 kbp from the closest genes (Table 2). Of the 13 additional SCR prevailing in control mice, mostly by three reads, more than half is positioned at a distance lower or close to 20 kbp from the closest genes (Table 3). It should also be mentioned that hundreds of reads are displayed by SCR positioned in chromosomes 6, 12, and 14, and that thousands of reads occur in SCR positioned in chromosomes 2 and 9. The latter reads are markedly more numerous in L1 with respect to C1, but not in L2 with regard to C2 (Table 4). In addition, all of them intersect unreliable regions present in the black list.

It is also of relevance that annotated genes closest to SCR shared by all mice (*n* = 1,005; Figure 4, bottom panels) modulate dendritic and synaptic activity (Table 5), most likely by acting on nuclear DNA transcription (Table 6), in agreement with the prompt BMD transfer to nuclei (Giuditta and Rutigliano, 2018). Overall, this suggests that BMD keeps encoding the adaptive modulations of brain synaptic activity elicited by learning, thereby updating memory. Further studies of the annotated genes closest to all SCR (*n* = 1,461; Supplementary Table S1) will improve our understanding of brain responses to the ever-changing environmental modifications.

The synaptic origin of BMD (Cefaliello et al., 2019; Prisco et al., 2019) and the identification of genes modulating synaptic activity (Tables 1–3, 5, 6 and Supplementary Table S1) that are closest to learning SCR and, more generally, to SCR shared by all mice indicates that BMD is retrotranscribed from RNA templates near presynaptic synapses and astroglial processes. In addition, the quick transfer of BMD to nuclei (Giuditta and Rutigliano, 2018) suggests that nuclear BMD from learning mice may be transcribed into novel RNA possibly inserted into blood exosomes reaching germ cells and the progeny (Spadafora, 2017). The marked increment in dsDNA breaks in promoters of early-response genes of mice exposed to a new environment (Madabhushi et al., 2015) is likely to facilitate BMD transfer to nuclei concurrently elicited by a learning experience. If verified by experimental tests, such view would support BMD further role in transferring brain adaptive responses to the progeny (Giuditta et al., 2017), a long predicted process of DNA renewal (Giuditta, 1982).

## Data Availability Statement

The datasets for this study can be found in https://www.ncbi.nlm.nih.gov/bioproject/608649 and https://www.ncbi.nlm.nih.gov/sra/PRJNA608649.

## Ethics Statement

The experimental procedures were reviewed and approved by the Animal Ethics Committee of the University of Campania “L. Vanvitelli” (Naples, Italy).

## Author Contributions

AG devised the experiment, interpreted the data and wrote the manuscript. AG and JC performed the training sessions.

## Conflict of Interest

The authors declare that the research was conducted in the absence of any commercial or financial relationships that could be construed as a potential conflict of interest.

## Footnotes

1 https://bedtools.readthedocs.io/en/latest/

2 https://www.r-project.org

3 https://bioconductor.org/packages/release/bioc/html/GenomicRanges.html

4 https://github.com/Boyle-Lab/Blacklist/tree/master/lists

5 https://bioconductor.org/packages/release/bioc/html/Rsubread.html

6 https://bioconductor.org/packages/release/bioc/html/ChIPpeakAnno.html

7 http://bioconductor.org/packages/release/data/annotation/html/TxDb.Mmusculus.UCSC.mm10.knownGene.html

8 https://bioconductor.org/packages/release/bioc/html/BSgenome.html

9 http://bioconductor.org/packages/release/data/annotation/html/BSgenome.Mmusculus.UCSC.mm10.html

10 https://cran.r-project.org/web/packages/gProfileR/index.html

11 https://www.nature.com/articles/s41598-019-45839-z
